# Homœopathy in Our Colleges

**Published:** 1887-06

**Authors:** G. W. Sherbino

**Affiliations:** Abilene, Texas


### HOMŒOPATHY IN OUR COLLEGES.

EDITOR OF THE HOMŒOPATHIC PHYSICIAN:

In the *Medical Advance* for April is a criticism on my article in the March number of THE HOMŒOPATHIC PHYSICIAN.

He says: "Possibly your article has two points to it—first, to decry our College, and the second, to boom another. Had you been present at the I. H. A. meeting at Saratoga last June, you would have been witness to a similar attempt—*i. e.*, booming one college at the expense of all the rest; but it was so palpable a mistake that it was quickly laid on the table, and the participants erased it from the proceedings."

At the I. H. A. meeting of 1886, a motion was made by Dr. E. A. Ballard, and seconded by Dr. P. P. Wells, to indorse the teachings of the Missouri College. Professor Kent, through unselfishness, moved to have the whole matter laid on the table, which was carried. Now this is what the editor means about booming one college at the expense of "all the rest." The matter was investigated and brought before the noble body of the I. H. A. by two gentlemen unconnected with the Missouri College; not for any special interest in the same, nor for the good of any *one man*, but for *any* and *every* one who will come and partake of the water of life. They are true to their principles—a condition which we believed was applicable to the editor of the *Advance*.

What's the matter with the *Advance*, anyhow? If there is one college that has an unusual amount of the teachings of the principles of Hahnemann, let's boom her up, boys.

The facts are, the *Advance* wants to boom one college with a bomb-shell—they could not get at it in any other way.

"How important it is that every one of our colleges should have at least one professor who has the manliness to give our students the teaching of Hahnemann and instruct them in the knowledge of our law and build their young minds up in the most holy faith of *Similia similibus curantur*."

This is what the *Advance* calls booming one college. N. B. "Every one"—not one, but "every one." If I had been booming one college, why did I not say so?

Once upon a time there was a man traveling. He fell among thieves. He was robbed, wounded, left on the ground to die. One passed him on the right and another on the left. Neither paid any attention to him. But you will remember one man came along and took compassion on him, took him to an inn, had his wounds dressed, and when he was able to leave paid the bill. And after years this same man, who was so mercifully treated, treasured up (for some cause or other) malice in his heart against the Good Samaritan.

A dark horse comes to the front as usual, and says: "I have a chance now to avenge myself. I can fetch 'em! I will give that fellow in Abilene a death-blow, but in an instant Sherb— steps aside, and the dagger goes past him into the walls of the St. Louis College. Any one doubting the truth, if they are diligent, they can find the knife, with the end battered, at the Recorder's office, where they keep such things as a memento.

You will hear from Texas again in August.

G. W. SHERBINO.

ABILENE, TEXAS, April 15th, 1887.

---

